# Development and validation of a risk index to predict kidney graft survival: the kidney transplant risk index

**DOI:** 10.1186/s12874-021-01319-5

**Published:** 2021-06-21

**Authors:** Sameera Senanayake, Sanjeewa Kularatna, Helen Healy, Nicholas Graves, Keshwar Baboolal, Matthew P. Sypek, Adrian Barnett

**Affiliations:** 1 Australian Centre for Health Services Innovation (AusHSI) and Centre for Healthcare Transformation, School of Public Health & Social Work, Queensland University of Technology (QUT), Brisbane, QLD Australia; 2 Australian Centre for Health Services Innovation, Queensland University of Technology, 60 Musk Ave, QLD 4059 Kelvin Grove, Australia; 3 Royal Brisbane and Women’s Hospital, Brisbane, Australia; 4 School of Medicine, University of Queensland, Brisbane, Australia; 5 Duke-NUS Medical School, Singapore, Singapore; 6 Australia and New Zealand Dialysis and Transplant (ANZDATA) Registry, Adelaide, South Australia Australia

**Keywords:** Risk prediction, Machine learning, Graft failure, Kidney transplant

## Abstract

**Background:**

Kidney graft failure risk prediction models assist evidence-based medical decision-making in clinical practice. Our objective was to develop and validate statistical and machine learning predictive models to predict death-censored graft failure following deceased donor kidney transplant, using time-to-event (survival) data in a large national dataset from Australia.

**Methods:**

Data included donor and recipient characteristics (n = 98) of 7,365 deceased donor transplants from January 1st, 2007 to December 31st, 2017 conducted in Australia. Seven variable selection methods were used to identify the most important independent variables included in the model. Predictive models were developed using: survival tree, random survival forest, survival support vector machine and Cox proportional regression. The models were trained using 70% of the data and validated using the rest of the data (30%). The model with best discriminatory power, assessed using concordance index (C-index) was chosen as the best model.

**Results:**

Two models, developed using cox regression and random survival forest, had the highest C-index (0.67) in discriminating death-censored graft failure. The best fitting Cox model used seven independent variables and showed moderate level of prediction accuracy (calibration).

**Conclusion:**

This index displays sufficient robustness to be used in pre-transplant decision making and may perform better than currently available tools.

**Supplementary Information:**

The online version contains supplementary material available at 10.1186/s12874-021-01319-5.

## Introduction

Kidney transplant offers better quality of life and superior survival compared to other kidney replacement therapy modalities [1]. However, health systems around the world struggle to bridge the increasing gap between the high demand for kidney transplants and limited supply. One strategy is directing kidney grafts to recipients with the greatest longevity, thereby reducing both the number of graft failures and the number of patients dying with a functioning graft [2]. Risk prediction models, predicting graft failure prior to transplantation, are clinical supports in the complex decision making of matching recipients with the greatest longevity and allografts with low risk of failure.

There are several kidney graft risk prediction models in the literature that have assisted evidence-based medical decision-making in clinical practice [3, 4]. The Kidney Donor Risk Index (KDRI) developed by Rao et al. in 2009 has widespread uptake in clinical decision making [3], and is used in the US Kidney Allocation System [5]. The C-index, which indicates a prediction model’s ability to discriminate longer surviving grafts from shorter surviving grafts, is however 0.62, a value denoting only reasonable discrimination. Novel approaches based on statistics or machine learning methods have the potential to yield more accurate predictions [6]. 

Machine learning has evolved rapidly over recent decades and is already applied to some areas of medical diagnostics [7]. A recent systematic review by our group highlighted the role of machine learning based risk prediction models in medical decision making, leading to more accurate kidney transplant outcome predictions [8]. Our review however found models, other than those developed in the United States, were commonly derived from sample sizes of fewer than 1,000 patients.

Furthermore, none of the machine learning models developed so far modelled the time-to-event (survival) [8]. Instead, most used the binary outcome of failure or not. However, a binary approach treats a graft that survives one year equally to a graft that fails at two years, vastly different outcomes for the patient and the health system. These models do not factor in loss to follow-up. Therefore, incorporating the dynamic of time to event into the prediction model produces clinically and economically important additional information [9].

Our objective was to develop and validate statistical and machine learning predictive models to predict graft failure following deceased donor kidney transplant, using time-to-event data in a large national dataset from Australia.

## Methods

The protocol of this study has been peer reviewed and published [10]. Briefly, three machine learning (Survival Tree[11], Random survival forest[12] and Survival support vector machine[13]) and one traditional regression (Cox regression[14]) models of time-to-event (survival time) were generated. This study is reported using the methodology of Transparent Reporting of a Multivariable Prediction Model for Individual Prognosis or Diagnosis (TRIPOD)[15].

### Study cohort

The data source was the Australia and New Zealand Dialysis and Transplant Registry (ANZDATA)[16]. It collects and reports the prevalence, incidence and outcomes of dialysis and kidney transplanted patients across Australia. The dataset contained donor and recipient characteristics of 7,365 kidney only deceased donor transplants from January 1st, 2007 to December 31st, 2017 conducted in Australia.

### Outcome

The primary outcome was time to graft failure starting from the transplantation date. Patients who died with a functioning graft were included and were right censored at their death date. Patients with a functioning graft at the end of the study period were right censored on December 31st, 2017. Sixty-five patients (0.9%) were lost to follow-up and were right censored at their last known follow-up date.

### Independent variables

Our aim was to develop a risk index for use in pre-transplant decision making, hence we used only the variables available before the transplantation and variables reported in ANZDATA across all patient groups. In total, 67 possible independent variables, both recipient and donor characteristics, were identified[17].

### Model development

**Fig. 1:**
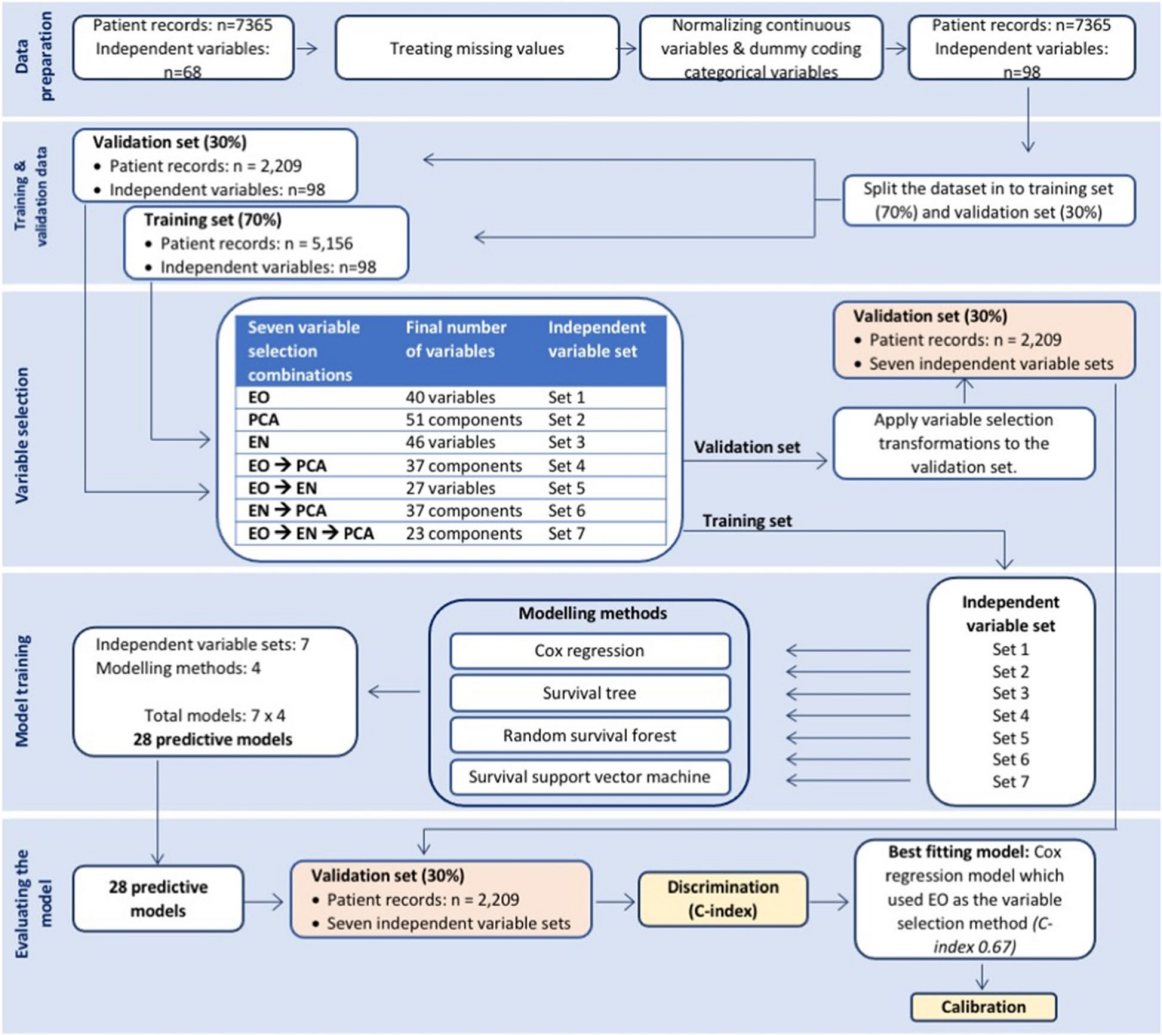
Model development and validation workflow. EO: Expert opinion; PCA: Principal component analysis; EN: Elastic net

Model development was a sequential process with the following five steps: data preparation, splitting the data set into training and validation datasets, variable selection, model training, and model evaluation (Fig. 1).

#### Step 1: Data preparation

Prior to model development, data were processed by: treating missing values, dummy coding categorical variables and scaling the continuous variables. The dataset had nearly 500,000 data points (7,365 patients × 67 independent variables) and 2.5% of the data points were missing. Most of the variables (64%) had less than 1% missing. For variables with missing values, multiple imputations were used for 14 categorical variables and 17 continuous variables with random hot deck and Classification and Regression Trees (CART) using the R package ‘simputation’[18] with the full dataset of 7,365 patients. Based on expert opinion, missing values of 13 categorical variables were assigned a separate category of “missing” to avoid the data being lost.

Numerical independent variables were normalized using min–max scaling, converting them to a similar scale to simplify the comparisons between variables[19]. Categorical variables were dummy coded into nominal categories. After dummy coding the total number of independent variables was 98.

#### Step 2: Training and validation data

The dataset was randomly divided into two parts: a training dataset and a validation dataset. The training set, used to train the four predictive models, contained 70% of the data (n = 5,156). The validation set (n = 2,209) was used to robustly test the predictive power of each model. Having a separate validation set provided more realistic estimates of the models’ prediction accuracy and helped avoid over-fitting.

#### Step 3: Variable selection

An important step in the model building process is the selection of a parsimonious set of predictor variables from the large set of available independent variables (n = 98). Too many independent variables in the model risks over-fitting, in turn reducing the predictive power[20].

Three methods were used to select the independent variables:

1. *Expert opinion:* Three experienced nephrologists reviewed the potential set of independent variables and indicated whether the variable had clinical significance. An agreement of at least two experts was considered adequate to include a variable into the model.
2. *Principal component analysis* [21] reduces the dimensionality of a dataset by transforming it to a smaller number of principal components based on the correlations between variables. This set of components ideally retains the majority of the variance and so does not lose information but does so using fewer variables. We used the number of principal components that retained 90% of the original variance.
3. *Elastic net* trades-off model fit and complexity to find a parsimonious model. It examines a range of models using penalties to avoid over-fitting that range from no penalty (Ridge regression – L_2_) to an extreme penalty (Lasso regression – L_1_) to find the ideal penalties trade-off point using cross-validation[22]. The L_1_ and L_2_ values which produced the lowest mean squared error during cross validation were used to fit the elastic net model.

These individual variable selection methods were applied alone and also in all possible combinations, e.g., expert opinion followed by elastic net. Therefore, a total of seven variable selection methods were used to generate seven different sets of independent variables.

#### Step 4: Model training

We used four approaches to model time-to-the primary event ie survival outcome.

Cox proportional regression[14]. This semi-parametric model is widely used to explore the relationship between outcomes like survival data and independent variables. After modelling the selected independent variables, the number of variables were further reduced by including only those that were statistically significant (p < 0.05). This made the model more parsimonious and also improved predictive power.

Survival Tree[11]. A survival tree is a tree-like structure, where leaves represent outcome variables, i.e. graft failure (1) or no graft failure (0), and branches are independent variables that influence the timing of the outcome. The complexity parameter was set to 0.00001 and the following two hyper-parameters were regularized until the optimal tree was created: the minimum number of samples that must exist in a node in order for a split to be attempted, and the number of competitor splits retained in the output.

Random survival forest (RSF) [12]. RSF is an ensemble method where numerous unpruned survival trees are developed via bootstrap aggregation[23, 24]. The ‘variable importance’ was set to “permutation” and the splitting rule to “log-rank”. The hyper-parameters, number of variables to possibly split at each node, number of trees and minimum number of nodes were regularised to achieve the lowest out-of-bag prediction error. ‘Variable importance’, a variable selection algorithm widely used in RSF, was used to avoid overfitting and to reduce the prediction error[25].

Survival support vector machine[13]. This uses hyperplanes to create classes of independent variables either with linearly (e.g. linear kernel function) or non-linearly separable data (e.g. polynomial kernel)[26, 27]. Based on the model’s performance, all support vector machine models were fitted using a linear kernel function with a ‘regression’ type survival support vector machine model.

The seven sets of independent variables were used to train and validate the four predictive models giving 28 results: seven variable selection methods × four predictive models. The predicted outcome for each of the four models was an index on an interval scale, which we label the Kidney Transplant Risk Index.

#### Step 5: Evaluating the models

We evaluated the models using methods proposed by Royston and Altman[28]. Model performance was evaluated using two metrics: discrimination and calibration. An index with good discrimination should have higher risk scores for higher risk patients and vice versa. Calibration measures the prediction accuracy as it compares the accuracy of the predicted survival from the index with the survival in the observed data[29]. For our study objective discrimination is more important than calibration, as our aim is to provide a guide to decision making that identifies relatively high and low risk patients[28]. Therefore the best model was chosen using the concordance index (C-index)[30], an index which evaluates the discriminative ability of a model. The C-index is defined as the fraction of pairs of patients where the patient who has a longer survival time also has a lower risk predicted score. The concordance range is between zero and one, with a higher value indicating better performance and 0.5 indicating discrimination by chance.

Appling Royston and Altman’s evaluation methods, the indices of the best fitting models were categorized into four groups at the 16^th^, 50^th^ and 84^th^ centiles to develop four prognostic groups: Good, Fairly good, Fairly poor and Poor. Use of unequal size groups improved discrimination of patients between the four groups and grouped patients with similar risk[28]. The survival of these four groups were compared using Kaplan–Meier plots, which ought, ideally, to show a large difference in survival between the four groups.

Calibration was visually assessed using the best fitting Cox model. Bootstrap resamples were used to estimate the bias-corrected predicted and observed mean survival at 3 and 5 years following transplantation[31]. Perfect agreement between the predicted and observed mean survival indicates a perfectly calibrated prediction model.

The best prediction model was compared with the predictive ability of the KDRI, which is the current model used by many clinical decision makers. The KDRI has 14 donor and transplant related variables and was developed using Cox regression to predict overall graft failure. The variables were selected using stepwise deletion of non-significant variables[3] and this model selection method has many limitations documented in the literature, including collinearity, p-values that are too small and confidence intervals that are too narrow[32].

The R programming language (version 3.6.0), with the libraries ‘survivalsvm’, ‘ranger’, ‘survival’ and ‘LTRCtrees’, was used to develop the predictive models[33].

### Ethics

Activities of the ANZDATA registry have been granted full ethics approval by the Royal Adelaide Hospital Human Research Ethics Committee. This study was granted ethics approval by the Queensland University of Technology.

## Results

### Baseline characteristics

The characteristics of the recipients and donors are in Table 1. The total study sample had 7,365 deceased donor kidney transplants performed from January 1^st^, 2007, to December 31^st^, 2017. The median age of donors was 52 years (inter-quartile range 41 to 60) and of the recipients was 47 years (inter-quartile range 32 to 58). The majority were males (63%). About 87% of the grafts were primary grafts.

**Table 1:** Baseline characteristics of recipients and donors

Characteristic	Value
Total	7365
*Recipient characteristics*	
Age in years (Median; IQR)	52 (41 – 60)
Sex (Male: Female)	63.2%: 36.8%
Diabetes mellitus n, (%)	1863 (25.3%)
Peripheral vascular disease n, (%)	558 (7.6%)
Hypertension n, (%)	1683 (22.9%)
Primary renal disease	
Diabetic Nephropathy	1355 (18.4%)
Glomerulonephritis	2886 (39.2%)
Hypertension	485 (6.6%)
Polycystic Disease	953 (12.9%)
Reflux Nephropathy	501 (6.8%)
Unknown	1185 (16.1%)
Months of haemo-dialysis among patients with any exposure to haemo-dialysis (n = 5833) (Median; IQR)	33.0 (14.3 – 60.7)
Months of peritoneal dialysis among patients with any exposure to peritoneal dialysis (n = 3621) (Median; IQR)	20.5 (10.2 – 36.7)
First graft	6422 (87.2%)
Graft failure n, (%)	693 (9.4%)
*Donor characteristics*	
Age (Median; IQR)	47 (32 – 58)
Diabetes mellitus n, (%)	450 (6.1%)
Hypertension n, (%)	1683 (22.9%)
Total ischaemia time in hours (Median; IQR)	11.0 (8.0 – 14.0)
Donation after brain death n, (%)	5815 (79.0%)

### Variable selection

There were 98 potential independent variables. Table 2 summaries the result of the three approaches to select a subset of independent variables that did not overfit, resulting in seven sets of independent variables. Expert opinion reduced the independent variables to 40 variables, while elastic net reduced it to 46 variables. Application of all three variable selection methods reduced the 98 potential variables to 23 principal components. Each of these seven sets of independent variables were used to train and test the models. During model building, independent variables were further reduced in cox and RSF by including only those that were statistically significant (p < 0.05) and including only those with positive ‘Variable importance’ (a variable selection algorithm used in RSF), respectively.

**Table 2:** Combinations of independent variable groups

Combination No	Order of variable selection	Final number of variables or components
Combination 1	EO	40 variables
Combination 2	PCA	51 components
Combination 3	EN	46 variables
Combination 4	EO PCA	37 components
Combination 5	EO EN	27 variables
Combination 6	EN PCA	37 components
Combination 7	EO EN PCA	23 components

*EO* Expert opinion; *PCA* Principal component analysis; *EN* Elastic net

### Model development and validation

The predictive performance of the models is compared in Table 3. Cox proportional regression and RSF outperformed the other two models (i.e. survival tree and support vector machine). The highest C-index (0.67) was from a Cox proportional regression model which used expert opinion as the variable selection method and RSF which used elastic net as the variable selection method. A C-index of 0.67 indicates moderate discriminative ability of death-censored graft failure. The discriminative ability of KDRI in discriminating death-censored graft failure was 0.53, a lower prediction ability than our two best models.

**Table 3:** C-index of the seven different variable selection methods and four predictive models. (More accurate models have a higher C-index. The joint two best indices are in bold)

No	Variable selection	Predictive models			
		**Cox**	**RSF**	**SVM**	**DT**
Combination 1	EO	**0.67**	0.66	0.58	0.60
Combination 2	PCA	0.65	0.60	0.65	0.55
Combination 3	EN	0.65	**0.67**	0.53	0.60
Combination 4	EO PCA	0.61	0.62	0.52	0.57
Combination 5	EO EN	0.66	0.61	0.61	0.57
Combination 6	EN PCA	0.64	0.65	0.56	0.61
Combination 7	EO EN PCA	0.64	0.63	0.62	0.60

*EO* Expert opinion; *PCA* Principal component analysis; *EN* Elastic net; *RSF* Random Survival Forrest; *SVM* Support Vector Machine; *DT* Decision Tree

The Cox model used 7 independent variables while the RSF used 20 variables (Table 4). Since the Cox model was able to produce the same discriminatory power with lower number of variables, it was considered as the best fitting model.

**Table 4:** Final set of independent variables in the best fitting Cox and RSF models

Mode	Number final variables	Variable names
Cox	7	*Donor variables (n* = *2)*
		Donor age, Donor hypertension
		*Recipient variables (n* = *5)*
		Age at transplant, Peripheral vascular disease^a^, Primary renal disease, Duration of peritoneal dialysis, Duration of haemodialysis
RSF	20	*Donor variables (n* = *10)*
		Donor age, DR locus 1, A locus 2, Height, Donor diabetes, Donor hypertension, Cause of death, Creatinine – terminal, Oliguria, Race
		*Recipient variables (n* = *10)*
		Age at transplant, HLA-DR mismatch, Pre-emptive transplant, Duration of peritoneal dialysis, Duration of haemodialysis, Primary renal disease, Smoking, Peripheral vascular disease, Age at starting renal replacement therapy, number of previous rejections

^a^Defined as presence of claudication symptoms

### Best fitting Cox model

**Fig. 2:**
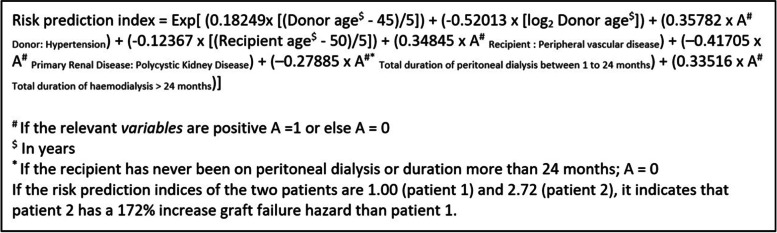
Calculation of the risk index using the Cox model. Peripheral vascular disease is defined as presence of claudication symptoms

As the donor age was a strong predictor of graft survival, a non-linear transformation of age (log base 2) was added into the model. This increased the C-index by only 0.003. We scaled the index to median donor (45 years) and recipient ages (50 years). The index of the Cox model is calculated as shown in Fig. 2.

A Weibull model, which assumes that the hazard is time-dependent[34], was also fitted as an alternative. However, the C-index reduced by 0.0014, not increasing discrimination, and so we kept the Cox model.

Donor hypertension (HR 1.43; 95%CI 1.16 to 1.76) increased the hazard while having polycystic kidney disease as the primary renal disease reduced the hazard (HR 0.66; 95%CI 0.48 to 0.91) (Table 5) of failure.

**Table 5:** Independent variables in the best fitted Cox model with their hazard ratios and 95% confidence

Variables in the Cox model		
	**Hazard ratio**	**95% Confidence interval**
***Donor variables***		
Age *(scaled to 5 years)*	1.20	1.12 to 1.28
Log_2_ of age	0.59	0.43 to 0.80
Donor Hypertension	1.43	1.16 to 1.80
***Recipient variables***		
Age at transplant *(scaled to 5 years)*	0.88	0.85 to 0.91
Peripheral vascular disease	1.41	1.03 to 1.93
Primary Renal Disease		
Polycystic Disease	0.66	0.48 to 0.91
Total duration of PD 1–24 months	0.75	0.61 to 0.94
Total duration of HD > 24 months	1.40	1.16 to 1.68

The distribution of the index over all patients shows that scores of risk groups, “Good” (< 16^th^ centile) and “Fairly good” (16^th^–50^th^ centile), have a narrow separation, whereas the other two categories (“Fairly poor” and “Poor”) are clearly separated (Supplementary Figure 1). This indicates that the Cox model does better at separating the higher risk groups.

**Fig. 3:**
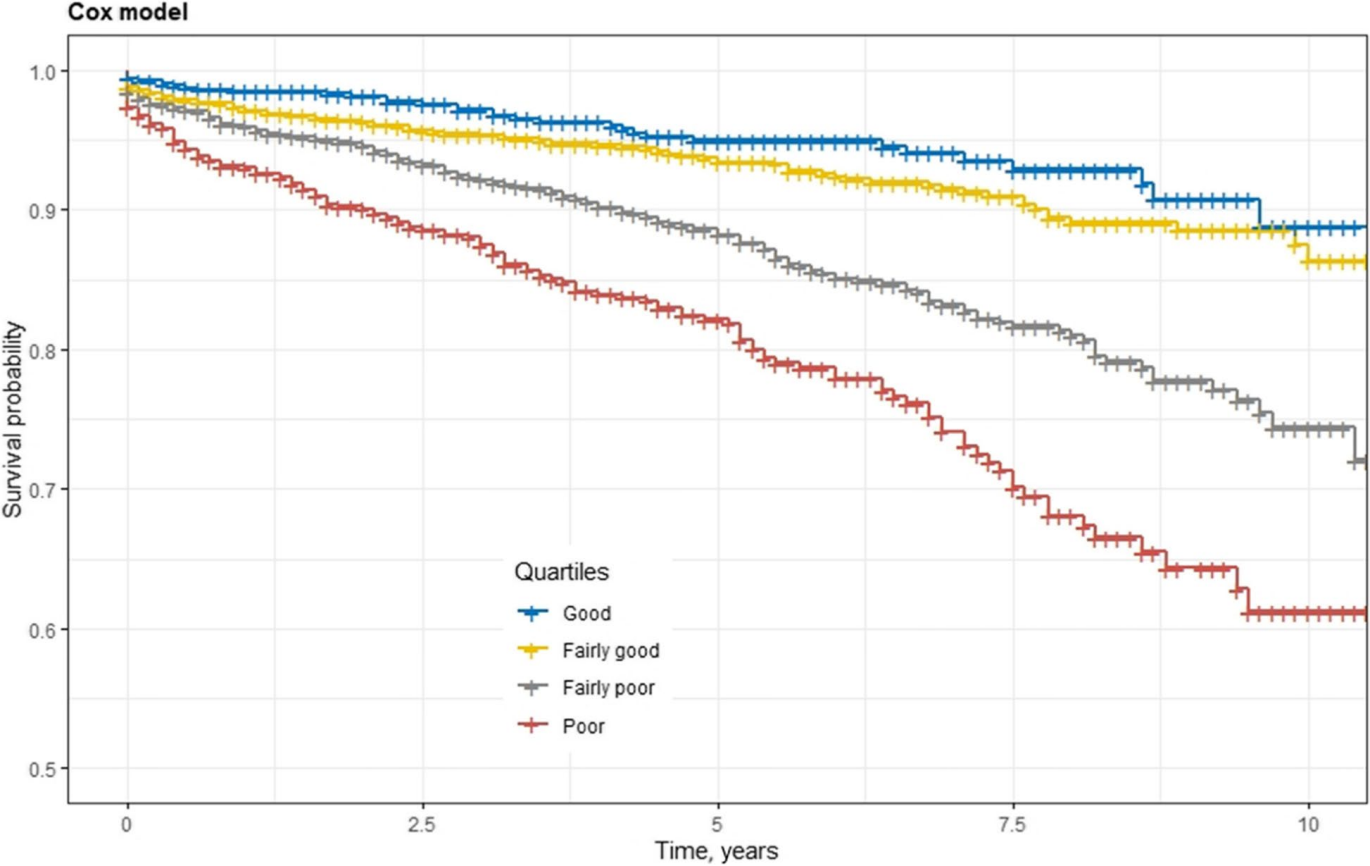
Kaplan–Meier survival curves indicating death-censored kidney graft failure by different risk prediction levels in the best fitting Cox model. The y-axis starts at a survival of 0.5 and not zero in order to more clearly show the separation between groups

The Cox model was able to discriminate the extreme categories of graft failure risk (Good vs Poor) with good discriminative power (C-index = 0.73). Discrimination between other groups was moderate (C-index > 0.6) (Table 6). Kaplan–Meier survival curves showing death-censored kidney graft failure for the four risk groups are in Fig. 3. As the risk groups move from “Good” to “Poor”, the survival curves demonstrate a marked increasing risk of graft failure. Furthermore, compared with the group “Good”, as the groups move from “Fairly good” to “Poor”, the hazard ratios increase in both training and validation datasets (Table 7). These results demonstrate that the index has good discriminatory power[28].

**Table 6:** Discriminative ability of different Kidney Transplant Risk Index prognostic groups by the best fitting Cox model

Risk categories	Cox model			
	**Good**	**Fairly good**	**Fairly poor**	**Poor**
**Good**				
**Fairly good**	0.62			
**Fairly poor**	0.64	0.61		
**Poor**	0.73	0.70	0.63

**Table 7:** Hazard ratios evaluated in the best fitting Cox model

	**Training set**		**Validation set**	
	**Hazard ratio**	**Standard error**	**Hazard ratio**	**Standard error**
HR: Fairly good vs Good	1.432	0.19	1.232	0.31
HR: Fairly poor vs Good	2.730	0.18	2.486	0.29
HR: Poor vs Good	4.580	0.18	5.499	0.29

**Fig. 4:**
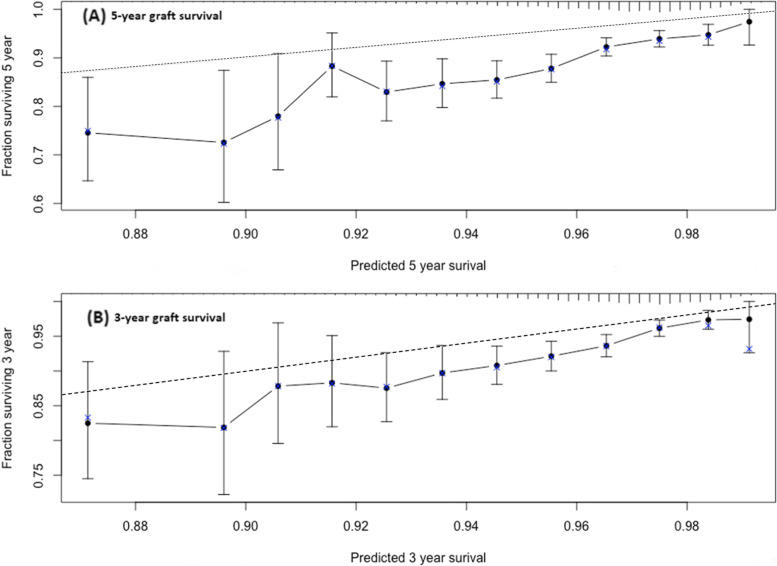
Mean predicted survival (dashed line) versus the mean actual survival at 3 years and 5 years following transplantation

The mean estimated survival compared with the mean actual survival at 3-years and 5-years is plotted in Fig. 4. In a perfectly calibrated model, data points would lie along the dashed line (perfect prediction line), indicating perfect prediction accuracy. The mean actual survival is consistently lower than the predicted survival at both 3 and 5 years. However, the gap between the prefect prediction line and the prediction line at both time periods reduces as the predicted survival increases. Overall, the Cox model shows moderate level of prediction accuracy.

## Discussion

Our study developed a risk prediction model to predict graft failure a priori, using a large sample of patients. We analysed four possible prediction models using statistical and machine learning methods. The best model was a Cox regression risk prediction model, which could predict death-censored graft failure with a moderate level of discrimination and prediction accuracy using only seven independent variables. The discriminatory power of the current index outperforms most of the currently available graft-failure risk prediction models.

The risk prediction model was developed to use in pre-transplant decision making (eg. kidney allocation), hence only the variables available before the transplantation were considered as independent variables. We used internal validation to create a parsimonious model because using a large number of independent variables can easily create poorly performing models that are not generalizability due to overfitting [35]. Stepwise variable selection, a commonly used variable selection method that was used to development the KDRI, is an unstable method that may create models that perform poorly in external validation [28]. Use of seven different variable selection combinations in the current study, identified by a combination of expert opinion and statistics, helped to identify the most important variables that explained most of the variance in the data. A parsimonious model results in an index that is easier to be use in a clinical setting. The final best Cox model has seven variables, fewer than the number of variables used in the most commonly used graft failure risk prediction models [3, 36].

Cox model outperformed the three machine learning methods used in the study. A review of literature indicates that the prediction accuracy provided mixed results when machine learning and traditional predictive methods are compared [8]. The current study used two tree-based machine learning methods and the poorer performance of these methods in our data may be an indication that the data does not have an underlying tree structure, where outcomes are determined by binary splits. Rather the risk of graft survival may be more dependent on continuous predictors, such as age.

Our model was developed to predict death-censored graft failure, whereas overall graft failure includes a combination of graft failure as well as death with a functioning graft. Knowledge of the survival of a given donor kidney is more important than the overall graft failure in pre-transplant decision making[2]. In our study, the C-index of death-censored graft failure was 0.67. Clayton et al. validated the US KDRI, using Australian data [2], and the C-index in discriminating death-censored graft failure was 0.63 which is lower discrimination than the results obtained here. However, inclusion of both transplant and recipient characteristics (total independent variables 24) in the KDRI increased the C-index of death-censored graft failure to 0.70 in the Clayton et al. study. These authors did not assess the calibration (prediction accuracy), which hinders a comprehensive comparison with the results of our study. Our best model has a C-index of 0.67 for just seven variables compared with a C-index of 0.70 for 24 variables in the latter, and clinicians may view this small increase in accuracy as not worth the increase in complexity. Prediction models with many variables are also more logistically difficult as they require more data to be collected and just one missing variable means the prediction cannot be estimated.

Furthermore, discriminatory power of the current index outperformed couple of other currently available indices, including KDRI as described earlier. Kasiske et al. (2010), developed an index with 11 donor and recipient variables, available before the transplantation, and it had a C-index of 0.649 [37]. A more recent index by Molnar et al., in 2018, had a C-index of 0.63 in discriminating high risk patients of graft failure. This index used 10 donor and recipient characteristics [38]. Therefore, the index described in this paper was able to achieve superior discriminatory power with fewer variables. However, we must consider whether a prediction using an index with a moderate discriminatory ability (C-index 0.67) is acceptable for the purpose of allocating kidneys, as the model is far from the perfect C-index of 1, meaning we cannot be sure that the predicted allocations will give the best outcomes. A false high index reading in the prediction model at the time of the transplant may discourage the clinician as well as the patient from accepting a donor kidney. This stigmatising effect of erroneously labelling a donor kidney as ‘marginal/low quality’ has already been documented [39].

Prediction of graft failure is a complex phenomenon, which involves donor characteristics, features related to donor organ retrieval, recipient characteristics, features related to the transplantation, and post-transplantation factors such as the use of immunosuppression drugs. Decisions related to kidney allocation, of course, have to be made prior to transplanting; therefore, transplant procedural and post-transplant factors are not available at the time of that initial decision making. Therefore, the source of the variability that is not accounted for in most of the currently available prediction models (as demonstrated by their only moderate level of discriminatory ability) may be transplant procedural or post-transplant-related factors. They may also be donor or recipient factors that are not routinely captured in databases, as well as unpredict stochastic events, leading to imperfect predictions. This means that a perfect C-index of 1 is likely impossible for a model using pre-transplantation factors. It is hard to know what the highest achievable C-index is, and this would require a separate modelling exercise that includes unverifiable assumptions about the importance of: 1) stochastic events and 2) unmeasured predictors.

The Cox model was able to discriminate the extreme categories of graft failure risk (Good vs Poor) with good discriminative power (C-index = 0.73), thus, the utility of the instrument among extreme categories of graft failure risk is superior compared to other risk categories. Therefore, limiting the use of the instrument only among these risk categories may produce superior results in kidney transplant decision making.

We have used robust internal validation, but external validation is an important step towards acceptance of a risk index into clinical decision making as assessing the performance of a model based on only internal validation may lead to an overly optimistic assessment of performance [28]. Furthermore, most clinicians may be unwilling to use a tool that has not been tested on different kidney populations. Hence, we propose that this index should be externally validated to assess generalizability prior to use in clinical practice. If the index demonstrates good external validity, the index has the potential to better fit donor to recipients, improving the current kidney allocation. Since this index has both donor and recipient features it can predict which donor-recipient match has the highest post-transplant survival, among available choices.

This study has several limitations. The predictive model used only the variables collected by ANZDATA, hence, we may have not included the complete risk profile of patients. We only used four methods and other machine learning methods that could model time-to-event information may have produced better results. However, machine learning models for survival data are not well developed, limiting our selection of the model types of best application [35].

## Conclusion

In summary the new index discriminates patients with higher risk of graft failure moderately well and makes graft failure predictions with a moderate level of accuracy. This promising new index is worth the next step of external validation to prove its use in clinical settings.

## Supplementary Information

Additional file 1: ** Supplementary figure 1** : Histogram of the index in the training and validation datasets of the best fitting Cox model.

## Data Availability

The datasets generated and/or analysed during the current study are not publicly available due to privacy and confidentiality agreements as well as other restrictions but are available from the corresponding author on reasonable request.

## Abbreviations

ANZDATA: Australia and New Zealand Dialysis and Transplant Registry ()
HR: Hazard ratio
KDRI: Kidney Donor Risk Index
RSF: Random survival forest
TRIPOD: Transparent Reporting of a Multivariable Prediction Model for Individual Prognosis or Diagnosis
