# Supplementary material for: Development and validation of a risk index to predict kidney graft survival: the kidney transplant risk index
Source: BMC Med Res Methodol. 2021 Jun 21;21:127. doi: 10.1186/s12874-021-01319-5 (PMC8215818; doi:10.1186/s12874-021-01319-5)
Supplement: Supplementary file 1 — Supplementary figure 1 : Histogram of the index in the training and validation datasets of the best fitting Cox model. [file 12874_2021_1319_MOESM1_ESM.pdf]

Supplementary material

Development and validation of a risk index to predict kidney graft survival: the Kidney Transplant Risk Index

Sameera Senanayake[1]; Sanjeewa Kularatna[1]; Helen Healy[2,3]; Nicholas Graves[4]; Keshwar Baboolal[2,3]; Matthew P. Sypek[5]; Adrian Barnett[1]

[1] Australian Centre for Health Services Innovation (AusHSI) and Centre for Healthcare Transformation, School of Public Health & Social Work, Queensland University of Technology (QUT), Brisbane, Queensland, Australia
[2] Royal Brisbane and Women's Hospital, Brisbane, Australia
[3] School of Medicine, University of Queensland, Australia
[4] Duke-NUS Medical School, Singapore
[5] Australia and New Zealand Dialysis and Transplant (ANZDATA) Registry, Adelaide, South Australia, Australia

Corresponding author

Sameera Senanayake
Australian Centre for Health Services Innovation,
Queensland University of Technology,
60 Musk Ave, Kelvin Grove, QLD 4059, Australia
s2.senanayake@qut.edu.au
+61450865361

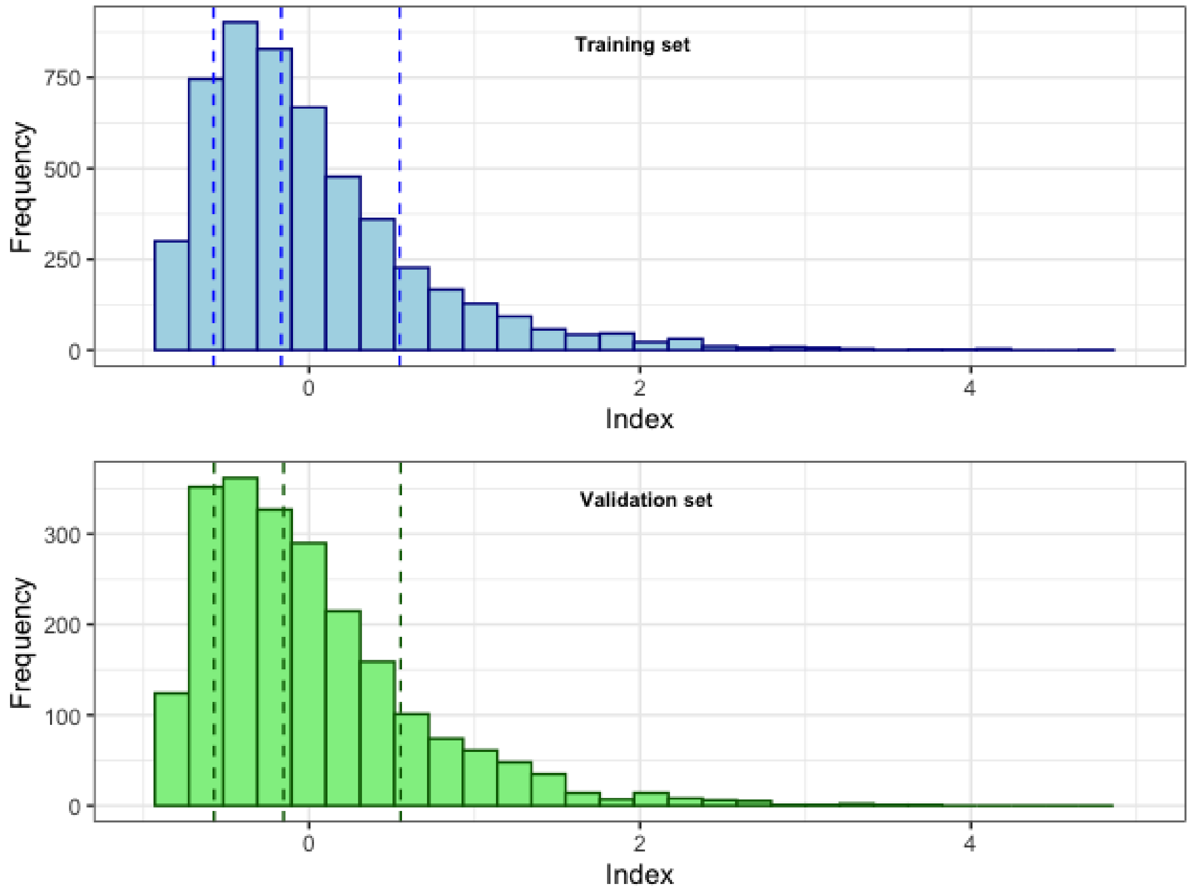

Supplementary figure 1 : Histogram of the index in the training and validation datasets of the best fitting Cox model.
The index was centered on the mean in the training dataset. The vertical lines show the 16th, 50th and 84th centiles of the index in each dataset.
